# Epidemiological factors associated with recent HIV infection among newly-diagnosed cases in Singapore, 2013–2017

**DOI:** 10.1186/s12889-021-10478-5

**Published:** 2021-03-02

**Authors:** Li Wei Ang, Carmen Low, Chen Seong Wong, Irving Charles Boudville, Matthias Paul Han Sim Toh, Sophia Archuleta, Vernon Jian Ming Lee, Yee Sin Leo, Angela Chow, Raymond Tzer-Pin Lin

**Affiliations:** 1 National Public Health and Epidemiology Unit, National Centre for Infectious Diseases, 16 Jalan Tan Tock Seng, Singapore, 308442 Singapore; 2 National Public Health Laboratory, National Centre for Infectious Diseases, Singapore, Singapore; 3 National HIV Programme, National Centre for Infectious Diseases, Singapore, Singapore; 4 Department of Infectious Diseases, Tan Tock Seng Hospital, Singapore, Singapore; 5 Yong Loo Lin School of Medicine, National University of Singapore, Singapore, Singapore; 6 Saw Swee Hock School of Public Health, National University of Singapore, Singapore, Singapore; 7 Division of Infectious Diseases, Department of Medicine, National University Hospital, National University Health System, Singapore, Singapore; 8 Communicable Diseases Division, Ministry of Health, Singapore, Singapore; 9 National Centre for Infectious Diseases, Singapore, Singapore; 10 Lee Kong Chian School of Medicine, Nanyang Technological University, Singapore, Singapore; 11 Department of Clinical Epidemiology, Office of Clinical Epidemiology, Analytics, and Knowledge (OCEAN), Tan Tock Seng Hospital, Singapore, Singapore; 12 Department of Laboratory Medicine, National University Hospital, National University Health System, Singapore, Singapore

**Keywords:** Recent HIV infection, Newly diagnosed, Epidemiology, Risk factors, HIV testing, Modifiable behaviors

## Abstract

**Background:**

Early diagnosis is crucial in securing optimal outcomes in the HIV care cascade. Recent HIV infection (RHI) serves as an indicator of early detection in the course of HIV infection. Surveillance of RHI is important in uncovering at-risk groups in which HIV transmission is ongoing. The study objectives are to estimate the proportion of RHI among persons newly-diagnosed in 2013–2017, and to elucidate epidemiological factors associated with RHI in Singapore.

**Methods:**

As part of the National HIV Molecular Surveillance Programme, residual plasma samples of treatment-naïve HIV-1 positive individuals were tested using the biotinylated peptide-capture enzyme immunoassay with a cutoff of normalized optical density ≤ 0.8 for evidence of RHI. A recent infection testing algorithm was applied for the classification of RHI. We identified risk factors associated with RHI using logistic regression analyses.

**Results:**

A total of 701 newly-diagnosed HIV-infected persons were included in the study. The median age at HIV diagnosis was 38 years (interquartile range, 28–51). The majority were men (94.2%), and sexual route was the predominant mode of HIV transmission (98.3%). Overall, 133/701 (19.0, 95% confidence interval [CI] 16.2–22.0%) were classified as RHI. The proportions of RHI in 2015 (31.1%) and 2017 (31.0%) were significantly higher than in 2014 (11.2%). A significantly higher proportion of men having sex with men (23.4, 95% CI 19.6–27.6%) had RHI compared with heterosexual men (11.1, 95% CI 7.6–15.9%). Independent factors associated with RHI were: age 15–24 years (adjusted odds ratio [aOR] 4.18, 95% CI 1.69–10.31) compared with ≥55 years; HIV diagnosis in 2015 (aOR 2.36, 95% CI 1.25–4.46) and 2017 (aOR 2.52, 95% CI 1.32–4.80) compared with 2013–2014; detection via voluntary testing (aOR 1.91, 95% CI 1.07–3.43) compared with medical care; and self-reported history of HIV test(s) prior to diagnosis (aOR 1.72, 95% CI 1.06–2.81).

**Conclusion:**

Although there appears to be an increasing trend towards early diagnosis, persons with RHI remain a minority in Singapore. The strong associations observed between modifiable behaviors (voluntary testing and HIV testing history) and RHI highlight the importance of increasing the accessibility to HIV testing for at-risk groups.

**Supplementary Information:**

The online version contains supplementary material available at 10.1186/s12889-021-10478-5.

## Introduction

Knowledge of HIV serostatus is an important element of HIV prevention and treatment efforts. Early diagnosis enhances the effectiveness of all subsequent steps in the cascade of HIV care, including initiation of combination antiretroviral therapy (ART) in the early phase of infection [1]. Immediate treatment is recommended for all HIV patients with detectable viremia regardless of CD4 count for better prognosis, and to reduce HIV transmission at the population level [2–4].

In Singapore, a cumulative total of 7982 HIV-infected Singapore residents had been notified to the National HIV Registry as of end-2017 [5]. The annual number of HIV notifications remained stable at an average of 450 in 2007–2017. The proportion of women diagnosed with HIV in Singapore was extremely low at 10% or less, in stark contrast to that of the Southeast Asian region where women constituted 37% of HIV diagnoses [6]. Sexual transmission accounted for 97% of all notifications. There were about 6900 (95% confidence interval [CI] 6650–7050) persons living with HIV in Singapore as of end-2014, and among them, 71.7% (95% CI 70.0–74.2%) had been diagnosed [7].

Despite the availability of effective highly active ART since 1996, a local study found that 54% of persons newly-diagnosed with HIV in 1996–2009 had late presentation to care, defined as having either a CD4 T-helper lymphocyte count (CD4 count) < 200 cells/mm^3^ at the time of presentation to care, or AIDS-defining conditions within 1 year of HIV diagnosis [8]. However, there are varying definitions for late diagnosis, which limits the comparability between studies [9, 10]. Some studies used a combination of laboratory-based definitions, such as CD4 count < 200 cells/mm^3^ [11] or < 350 cells/mm^3^ [12], and a clinical definition based on the occurrence of an AIDS-defining event in 3 months [11], 6 months [12, 13] or 1 year [14, 15] following HIV diagnosis. Although these definitions indicate the stage of disease progression at the time of diagnosis in relation to the optimal time for commencement of treatment, they do not constitute a direct measure of time from HIV infection [16].

Recent HIV infection (RHI) classification can be used as an indicator for early diagnosis, as recent infection generally refers to the phase up to 6 months after acute infection during which detectable anti-HIV-1 antibodies develop [17]. RHI implies ongoing transmission, and ascertainment of current HIV transmission patterns provides insights to guide preventive and interventional strategies targeted at high-risk individuals. The aims of this retrospective study were to estimate the proportion of RHI and elucidate epidemiological factors associated with RHI in Singapore.

## Methods

### Study population

HIV is a legally notifiable disease in Singapore. The HIV notification system is supplemented with additional information obtained through review of medical case notes and interviews with the cases. The information collected on all HIV cases includes socio-demographic characteristics, the first CD4 count, mode of detection and exposure factors.

To better monitor the dynamics of HIV transmission in Singapore, the National Public Health Laboratory (NPHL) commenced detection of RHI on residual plasma samples from HIV cases since 2013, as part of the National HIV Molecular Surveillance Programme under the Infectious Diseases Act (IDA) [18]. The proportion of RHI in newly-diagnosed HIV cases can be estimated using serological assays to measure the level of HIV-1-specific antibodies out of total immunoglobulin (IgG), which increases with time since infection [19, 20]. To further increase the specificity of the result, a recent infection testing algorithm (RITA), taking CD4 counts and supplementary clinical information into consideration to classify an HIV infection as recent or long-term, was applied as recommended by the World Health Organization (WHO) guidelines [21]. The main advantage of such an approach in determining the evidence of RHI is that a single sample can be taken at the time of HIV diagnosis without the need for follow-up, unlike with cohort studies.

Test results from NPHL were linked to the National HIV Registry using unique personal identifiers tagged to the samples. Personal identifiers were permanently removed from the merged database prior to statistical analysis.

Residual plasma samples of individuals whose HIV infection had been confirmed by Western blot assay were collected from public acute-care tertiary hospitals for the National HIV Molecular Surveillance Programme. We excluded HIV cases notified to the National HIV Registry from this study if they met one or more of the following criteria: unavailability of CD4 count at diagnosis; presence of an AIDS-defining illness at the time of diagnosis; commencement of ART prior to specimen collection. The testing for RHI was confined to plasma samples drawn from treatment-naïve individuals within 12 months of HIV diagnosis in 2013–2017.

### Laboratory methods

The biotinylated peptide-capture enzyme immunoassay (BED-CEIA) assay (Sedia Biosciences Corp, Portland, USA) was performed according to the manufacturer’s instructions on frozen residual plasma [22]. This assay measures the proportion of HIV-1 specific immunoglobulin (IgG) relative to total IgG against an internal calibrator specimen. It detects increasing proportion of HIV-1 IgG following seroconversion [23, 24].

Briefly, human antibodies including HIV-specific antibodies were captured in the solid phase of the microplate. After incubation (60 min, 37 °C) and washing, the custom biotinylated peptide (BED) that includes divergent immunodominant gp41 sequences from all HIV-1 (group M) subtypes and recombinants, was added (60 min, 37 °C). After washing, the plate was incubated for 90 min with streptavidin-peroxidase. Following another around of washes, tetramethylbenzidine (TMB) was added for 15 min at room temperature. The reaction was stopped by the addition of a stopping solution (1 N sulfuric acid) and the optical density (OD) was read at 450 nm with a spectrophotometer.

Results were reported as normalized OD (OD-n) units, calculated by dividing the OD value of sample or median OD of controls by the median OD of the calibrator. All samples with OD-n ≤ 1.2 were tested in triplicates to confirm whether it was a recent or long-term infection. A final OD-n cut-off of < 0.8 was used to distinguish recent from long-term infection. This threshold corresponds to a mean recency period of 197 days (95% confidence interval [CI] 173–220) [19]. We applied RITA to individuals whose samples returned results indicating evidence of RHI. Individuals were reclassified as having non-RHI (NRHI) if they had CD4 count < 200 cells/mm^3^ [25].

### Statistical analysis

We calculated the 95% CI for binomial proportions using Wilson’s method. We compared individuals with RHI and those with NRHI using the Chi-square test or Fisher’s exact test for categorical variables, and Mann-Whitney U test for continuous variables.

The main outcome was whether an individual had RHI or NRHI. Crude and adjusted odds ratio were calculated using logistic regression analyses. Multivariable analysis was used to determine independent factors for RHI. Variables with *p* < 0.10 from univariable regression analyses were entered as initial candidates using backward stepwise selection process, and covariates with *p* < 0.05 were retained in the final multivariable model.

For variables with missing data proportion less than 30%, we used missForest package (version 1.4) of R, an iterative non-parametric method, to impute the missing values. This random forest-based method produces a single imputed dataset without setting aside test data or performing cross validations [26]. The proportion of missing data ranged from 0.4 to 8.4%. Sensitivity analyses were performed to assess the robustness of our findings by using listwise deletion for missing data of independent variables in the multivariable logistic regression model. We also inserted an “unknown” category for variables with missing data when determining the independent risk factors associated with RHI.

All *p* values reported were 2-sided and statistical significance was taken as *p* < 0.05. Statistical analyses were performed using R version 3.6.1 (R Foundation for Statistical Computing, Vienna, Austria) and Stata version 16 (StataCorp, College Station, TX).

## Results

**Fig. 1:**
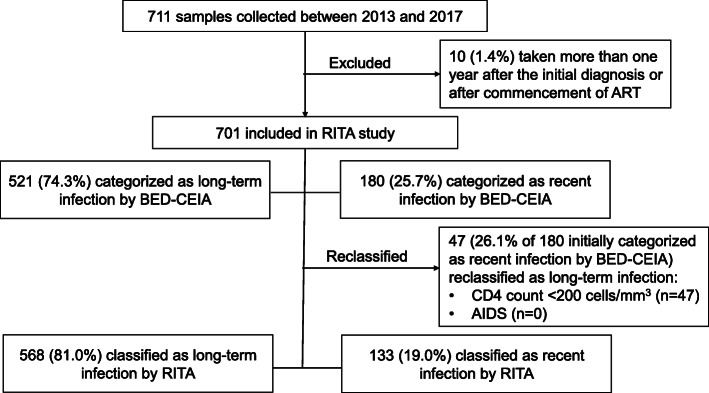
Flowchart of samples included in analyses and classified according to the recent infection testing algorithm (RITA), 2013–2017. ART: antiretroviral therapy

NPHL collected 711 samples for the National HIV Molecular Surveillance Programme, of which 10 (1.4%) did not meet the inclusion criteria for this study (Fig. 1). A total of 701 newly-diagnosed HIV-infected individuals were included in the analysis, and they constituted 31.8% of all HIV cases notified to the National HIV Registry in 2013–2017.

### Characteristics of study population

The median age at HIV diagnosis was 38 years (interquartile range [IQR], 28–51). The majority of the study population were male (94.2%), Chinese (72.0%), had never been married (72.8%), had attained education at post-secondary level (75.3%), and worked in professional/managerial positions or administrative/service-oriented sectors (62.6%) (Table 1). The main reasons for the current HIV test were medical care (38.1%), routine programmatic screening (31.8%), and voluntary testing (23.3%). Three in five newly-diagnosed HIV infections were attributed to homosexual/bisexual transmission (61.6%), and another 36.7% were accounted for by heterosexual contact. About half (52.5%) of the cases had undergone HIV test(s) prior to their diagnosis. Over two-thirds (65.0%) reported having regular and casual contacts only as sexual partners.

**Table 1:** Characteristics (%) of newly-diagnosed HIV-positive individuals included in the RITA study, and all cases notified to the National HIV Registry, 2013–2017

Characteristic	All HIV notifications (***N*** = 2207)	Included in RITA study			
		Total (***N*** = 701)	RHI (***N*** = 133)	NRHI (***N*** = 568)	***P***-value§
	n (%)	n (%)	n (%)	n (%)	
Age at HIV diagnosis (years)					< 0.0005
0–14	2 (0.1)	0 (0.0)	0 (0.0)	0 (0.0)	
15–24	220 (10.0)	96 (13.7)	34 (25.6)	62 (10.9)	
25–34	535 (24.2)	186 (26.5)	40 (30.1)	146 (25.7)	
35–44	572 (25.9)	157 (22.4)	30 (22.6)	127 (22.4)	
45–54	492 (22.3)	145 (20.7)	21 (15.8)	124 (21.8)	
55–64	285 (12.9)	87 (12.4)	7 (5.3)	80 (14.1)	
≥ 65	101 (4.6)	30 (4.3)	1 (0.8)	29 (5.1)	
Gender					0.840
Male	2061 (93.4)	660 (94.2)	126 (94.7)	534 (94.0)	
Female	146 (6.6)	41 (5.8)	7 (5.3)	34 (6.0)	
Ethnic group					0.695
Chinese	1538 (69.7)	505 (72.0)	97 (72.9)	408 (71.8)	
Malay	426 (19.3)	135 (19.3)	22 (16.5)	113 (19.9)	
Indian	141 (6.4)	41 (5.8)	10 (7.5)	31 (5.5)	
Others	102 (4.6)	20 (2.9)	4 (3.0)	16 (2.8)	
Marital status					< 0.0005
Never married	1522 (69.0)	510 (72.8)	116 (87.2)	394 (69.4)	
Married	472 (21.4)	125 (17.8)	15 (11.3)	110 (19.4)	
Separated/Divorced/Widowed	213 (9.7)	66 (9.4)	2 (1.5)	64 (11.3)	
Educational level					0.139
No formal /Primary	124 (5.6)	36 (5.1)	4 (3.0)	32 (5.6)	
Secondary	185 (8.4)	46 (6.6)	12 (9.0)	34 (6.0)	
Post-secondary	1617 (73.3)	528 (75.3)	93 (69.9)	435 (76.6)	
Tertiary	273 (12.4)	88 (12.6)	23 (17.3)	65 (11.4)	
Unknown	8 (0.4)	3 (0.4)	1 (0.8)	2 (0.4)	
Occupational type					0.048
Professional/executive	436 (19.8)	123 (17.5)	25 (18.8)	98 (17.3)	
Administrative/service-oriented	888 (40.2)	316 (45.1)	62 (46.6)	254 (44.7)	
Blue-collar worker	275 (12.5)	98 (14.0)	13 (9.8)	85 (15.0)	
Unemployed	80 (3.6)	16 (2.3)	0 (0.0)	16 (2.8)	
Others	290 (13.1)	101 (14.4)	27 (20.3)	74 (13.0)	
Unknown	238 (10.8)	47 (6.7)	6 (4.5)	41 (7.2)	
Year of HIV diagnosis					0.009
2013	454 (20.6)	49 (7.0)	4 (3.0)	45 (7.9)	
2014	456 (20.7)	116 (16.5)	13 (9.8)	103 (18.1)	
2015	455 (20.6)	166 (23.7)	40 (30.1)	126 (22.2)	
2016	408 (18.5)	214 (30.5)	39 (29.3)	175 (30.8)	
2017	434 (19.7)	156 (22.3)	37 (27.8)	119 (21.0)	
Reason for HIV testing					< 0.0005
Medical care	1046 (47.4)	267 (38.1)	27 (20.3)	240 (42.3)	
Voluntary testing	432 (19.6)	163 (23.3)	45 (33.8)	118 (20.8)	
Routine programmatic screening†	587 (26.6)	223 (31.8)	51 (38.3)	172 (30.3)	
Others	142 (6.4)	48 (6.8)	10 (7.5)	38 (6.7)	
Mode of HIV transmission					0.002
Heterosexual	881 (39.9)	257 (36.7)	31 (23.3)	226 (39.8)	
Homosexual/bisexual	1241 (56.2)	432 (61.6)	101 (75.9)	331 (58.3)	
IDU	13 (0.6)	2 (0.3)	0 (0.0)	2 (0.4)	
Others	9 (0.4)	1 (0.1)	0 (0.0)	1 (0.2)	
Unknown	63 (2.9)	9 (1.3)	1 (0.8)	8 (1.4)	
Ever tested for HIV prior to positive diagnosis					< 0.0005
Yes	1084 (49.1)	368 (52.5)	92 (69.2)	276 (48.6)	
No	837 (37.9)	274 (39.1)	31 (23.3)	243 (42.8)	
Unknown	286 (13.0)	59 (8.4)	10 (7.5)	49 (8.6)	
Type of sexual partners					0.056
Regular only	236 (10.7)	61 (8.7)	11 (8.3)	50 (8.8)	
Regular & casual only	1347 (61.0)	456 (65.0)	99 (74.4)	357 (62.9)	
Sex workers & social escorts	542 (24.6)	170 (24.3)	22 (16.5)	148 (26.1)	
Unknown	82 (3.7)	14 (2.0)	1 (0.8)	13 (2.3)	

*RHI* Recent HIV infection, *NRHI* Non-recent HIV infection, *RITA* Recent infection testing algorithm; *IDU* Intravenous drug use

† Routine programmatic HIV screening includes screening programmes for persons with sexually transmitted infections, hospital inpatients and those identified through contact tracing

§ *P*-value is for the comparison between RHI group (*N* = 133) and NRHI group (*N* = 568)

The socio-demographic and epidemiological characteristics of the 701 newly-diagnosed HIV-positive individuals in our study were broadly similar to the 2207 cases notified to the National HIV Registry during the five-year period (Table 1). There might have been an over-representation of HIV-positive individuals who acquired infection through sexual contact among men having sex with men (MSM) in our study (61.6%), when compared with that of all HIV notifications (56.2%).

### Proportion of recent HIV infection

Of the 701 residual plasma samples tested, 180 (25.7%) were initially classified as recently infected by reactivity on the BED-CEIA. Of these 180 individuals, 47 had low CD4 count (< 200 cells/mm^3^) and they were therefore reclassified as having long-term infection according to RITA, leaving 133 individuals classified as recently infected (Fig. 1). None of these 133 individuals had an AIDS-defining illness at diagnosis, hence no further reclassification was needed. Overall, about one-fifth (19.0, 95% CI 16.2–22.0%) of the newly-diagnosed, treatment-naïve HIV-positive individuals had RHI.

**Fig. 2:**
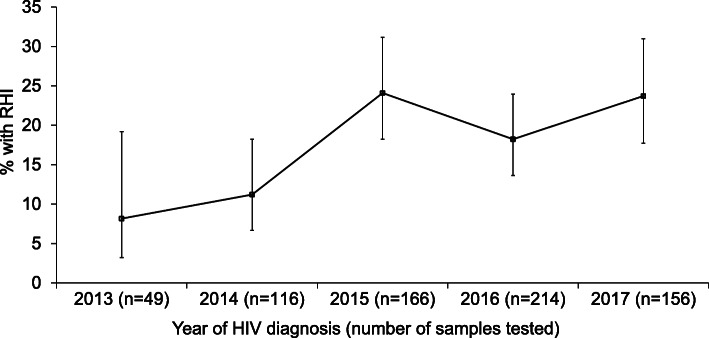
Proportion of recent HIV infection among newly-diagnosed HIV-positive individuals included in the RITA study, 2013–2017. The error bars indicate 95% confidence interval

**Fig. 3:**
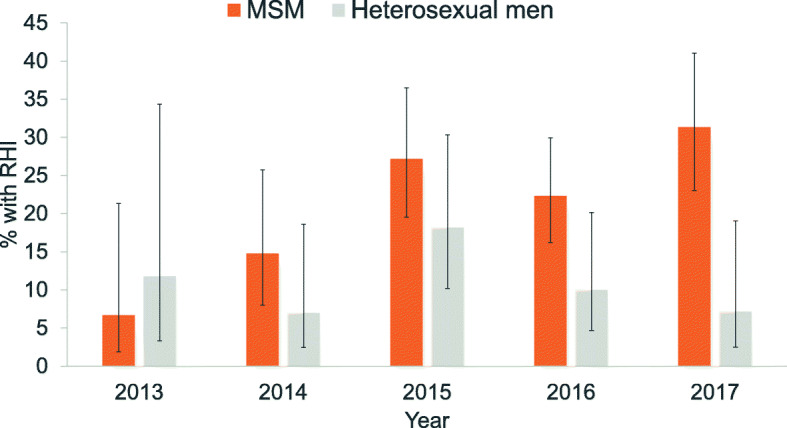
Proportion of recent HIV infection among newly-diagnosed HIV-positive men who had sex with men (MSM) and men infected with HIV via heterosexual transmission included in the RITA study, 2013–2017. The error bars indicate 95% confidence interval

The proportions of RHI in 2015 (31.1%) and 2017 (31.0%) were significantly higher than in 2014 (11.2%)(*p* < 0.01) (Fig. 2). Over the five-year study period, a significantly higher proportion of MSM (23.4, 95% CI 19.6–27.6%) had RHI compared with heterosexual men (11.1, 95% CI 7.6–15.9%). Stratification by year of HIV diagnosis revealed significant differences between these two exposure risk groups in 2016 and 2017 (Fig. 3).

### Factors associated with recent HIV infection

Recently infected individuals were younger than those with long-term infection; the median age was 32 years (IQR 24–44) in cases with RHI and 36 years (IQR 29–52) among those with NRHI (*p* < 0.0005). Individuals aged 15–24 at HIV diagnosis constituted a significantly higher proportion of those with RHI, when compared with NRHI (25.6% vs 10.9%), whereas those aged ≥55 years made up a lower proportion (6.0% vs 19.2%) (Table 1). A significantly higher proportion of individuals classified as having RHI were never married (87.2% vs 69.4%), diagnosed in later years of 2015–2017 (87.2% vs 73.9%), detected via voluntary testing (33.8% vs 20.8%), infected via homosexual/bisexual mode of transmission (75.9% vs 58.3%), and had history of HIV test(s) prior to diagnosis (69.2% vs 48.6%).

Univariable logistic regression analyses revealed that age group, marital status, year of diagnosis, reasons for current HIV test, mode of HIV transmission and whether the individual had been tested for HIV prior to positive HIV diagnosis were epidemiological factors associated with RHI (Table 2).

**Table 2:** Proportion and odds ratios of factors for recent HIV infection among newly-diagnosed HIV-positive individuals included in the RITA study, 2013–2017

Characteristic	% of RHI	Univariable model			Multivariable model^b^		
		cOR	(95% CI)	***P*** value	aOR	(95% CI)	***P*** value
Age at diagnosis (years)							
15–24	35.4	7.47	(3.26–17.15)	< 0.0005	**4.18**	**(1.69–10.31)**	**0.002**
25–34	21.5	3.73	(1.68–8.30)	0.001	2.19	(0.93–5.16)	0.073
35–44	19.1	3.22	(1.42–7.31)	0.005	2.01	(0.84–4.77)	0.115
45–54	14.5	2.31	(0.98–5.42)	0.055	1.83	(0.76–4.40)	0.176
≥ 55	6.8	1.00	Referent		1.00	Referent	
Gender							
Male	19.1	1.15	(0.50–2.64)	0.749			
Female	17.1	1.00	Referent				
Ethnic group							
Chinese	19.2	1.00	Referent				
Malay	16.3	0.82	(0.49–1.36)	0.440			
Indian & others	23.0	1.25	(0.66–2.37)	0.488			
Marital status							
Never married	8.2	1.00	Referent				
Married	11.2	0.46	(0.26–0.83)	0.009			
Separated/Divorced/Widowed	24.1	0.11	(0.03–0.44)	0.002			
Educational level^a^							
No formal/Primary	11.1	1.00	Referent				
Secondary	26.1	2.82	(0.83–9.66)	0.098			
Post-secondary	17.7	1.72	(0.60–4.99)	0.315			
Tertiary	25.8	2.79	(0.89–8.74)	0.079			
Occupational type^a^							
Professional/executive	20.2	1.00	Referent				
Administrative/service-oriented	19.5	0.96	(0.58–1.59)	0.867			
Blue-collar worker	12.3	0.55	(0.27–1.14)	0.109			
Unemployed & others	22.0	1.12	(0.61–2.04)	0.711			
Year of HIV diagnosis							
2013–2014	10.3	1.00	Referent		1.00	Referent	
2015	24.1	2.76	(1.49–5.11)	0.001	**2.36**	**(1.25–4.46)**	**0.008**
2016	18.2	1.94	(1.05–3.57)	0.033	1.69	(0.89–3.20)	0.107
2017	23.7	2.71	(1.45–5.05)	0.002	**2.52**	**(1.32–4.80)**	**0.005**
Reason for HIV testing							
Medical care	10.1	1.00	Referent		1.00	Referent	
Voluntary testing	27.6	3.39	(2.00–5.73)	< 0.0005	**1.91**	**(1.07–3.43)**	**0.029**
Routine programmatic screening^c^	22.9	2.64	(1.59–4.37)	< 0.0005	1.62	(0.93–2.83)	0.090
Others	20.8	2.34	(1.05–5.22)	0.038	1.79	(0.77–4.16)	0.174
Mode of HIV transmission^a^							
Heterosexual	11.9	1.00	Referent				
Homosexual/bisexual	23.2	2.24	(1.45–3.46)	< 0.0005			
IDU & others	25.0	2.47	(0.25–24.52)	0.439			
Ever tested for HIV prior to positive diagnosis^a^							
Yes	24.9	2.69	(1.75–4.12)	< 0.0005	**1.72**	**(1.06–2.81)**	**0.028**
No	11.0	1.00	Referent		1.00	Referent	
Type of sexual partners^a^							
Regular only	17.5	1.00	Referent				
Regular and casual only	21.5	1.30	(0.65–2.57)	0.461			
Sex workers & social escorts	12.7	0.69	(0.31–1.52)	0.355			

*cOR* Crude odds ratio; *aOR* Adjusted odds ratio

*RHI* Recent HIV infection, *RITA* Recent infection testing algorithm; *IDU* Intravenous drug use

^a^ Missing data were imputed

^b^ Variables in the final multivariable logistic regression model were age at HIV diagnosis, year of HIV diagnosis, mode of detection, and whether the person had previous HIV test(s). Significant associations in the multivariable model were highlighted in bold

^c^ Routine programmatic HIV screening includes screening programmes for persons with sexually transmitted infections, hospital inpatients and those identified through contact tracing

On multivariable logistic regression analysis, risk factors independently associated with RHI were: age 15–24 years (adjusted odds ratio [aOR] 4.18, 95% CI 1.69–10.31) compared with those ≥55 years, HIV diagnosis in 2015 (aOR 2.36, 95% CI 1.25–4.46) and 2017 (aOR 2.52, 95% CI 1.32–4.80) compared with 2013–2014, detection via voluntary testing (aOR 1.91, 95% CI 1.07–3.43) compared with routine medical care, and self-reported history of HIV testing prior to diagnosis (aOR 1.72, 95% CI 1.06–2.81) (Table 2).

We obtained similar results (not shown) when an “unknown” category was included for variables with missing data in the multivariable logistic regression model. In complete case analysis, detection via voluntary testing was no longer statistically significant (*p* = 0.078) in the multivariable model.

## Discussion

Among newly-diagnosed HIV-positive individuals who had yet to receive ART, nearly one-fifth had acquired their HIV infection within 6 months of diagnosis. We found that the majority of HIV-infected persons were diagnosed later in the course of the disease, which underlines the problem of delayed HIV diagnosis in Singapore. The annual proportion of late presentation among all newly-diagnosed cases notified to the National HIV Registry has remained considerably high, ranging from 40.4 to 48.7% in 2013–2017.

The overall proportion of RHI among newly-diagnosed HIV cases in our study (19.0%) was lower than that of Tokyo, Japan (38.6%) [27], Taiwan (43.8%) [28], and Australia (25.0% in 2017) [29] (Table S1). In Europe, the estimated proportion of RHI ranged from 14.7 to 47.3% in developed countries [16, 30–38], based on one of the three assays (avidity index assay, detuned assay and BED assay) in different time periods (Table S1). In the United States, 20% of patients newly-diagnosed with HIV in 1997–2001 had acquired their infection within 6 months of diagnosis [39]. We found serological evidence of RHI in 23.4% of newly-diagnosed HIV-positive MSM, which was lower than that of China (41.9%) [40] and the state of Victoria, Australia (35.8%) [41]. However, some caution should be exercised in the interpretation and comparison of the proportion of RHI, as it largely depends on testing patterns of the at-risk population and underlying pattern of HIV incidence [30].

A higher proportion of RHI implies either a higher frequency of HIV testing and/or HIV incidence in certain risk groups. In this study, we observed higher overall proportions of RHI in 2015 and 2017 (Fig. 2), which corresponded to the trend observed in MSM (Fig. 3). The overall proportion of RHI among HIV-infected MSM (23.4%) was double that of men infected with HIV via heterosexual transmission (11.1%). This is corroborated by the national notification data in which a significantly higher proportion of late presenters were among cases who acquired HIV infection through heterosexual contact compared with those infected via MSM contact [8]. MSM in Singapore are known to undergo more frequent HIV testing, possibly due to their recognition of the importance of regular screening and/or an indication that they are at higher risk of infection after a risk exposure. These are potentially the result of ongoing targeted sexual health messaging campaigns in the country. An outreach HIV testing project conducted by Action for AIDS (AfA), a local non-governmental HIV/AIDS community-based organization, at venues frequented by MSM in Singapore found that there were fewer first-time testers in 2013 than in previous years, and about half of the MSM had been tested in the 12 months prior to the survey [42]. The proportion of RHI among newly-diagnosed HIV-positive MSM had also increased in the United Kingdom [43] and Germany [44].

Independent risk factors associated with RHI identified in studies conducted in many European countries [16, 30, 35–38], and the United States [39] include younger age, MSM, high economic status, those who underwent testing after a risk exposure, higher frequency of HIV testing, more sexual partners and history of diagnosis of sexually transmitted infections (STIs) (Table S1). In our study, younger age, HIV diagnosis in more recent years, detection via voluntary testing, and history of HIV test(s) prior to positive diagnosis were independent factors associated with RHI (Table 2).

Compared with newly-diagnosed HIV-positive individuals aged ≥55 years, those aged 15–24 years were more likely to have been infected recently. The proportion of RHI declined with age, from 35.4% in newly-diagnosed cases aged 15–24 years to 6.8% in those aged ≥55 years (*p* < 0.0005) (Table 2). On the other hand, the proportion of late presentation among HIV cases is known to increase with age [8]. Data from AfA’s anonymous testing and counselling service indicated that the majority of clients who get tested are 20–39 years of age (81%) [45], suggesting more frequent HIV testing among younger adults than older individuals.

Detection via voluntary testing was an independent factor associated with higher likelihood of RHI. The decision to be voluntarily tested for HIV is usually based on awareness of testing benefits and perception of the risk of recent HIV exposure. A local study on male HIV cases infected via the sexual route and diagnosed in 1985–2007 found that MSM were more likely to undergo voluntary testing than heterosexual men [46].

Self-reported history of HIV testing prior to positive diagnosis was an independent factor for RHI. Our study revealed that previous HIV testing was reported in 69.2% of the cases with RHI, significantly higher than the 48.6% among those with NRHI (Table 1). This is to be expected, as a recent infection is more likely to be picked up among repeat testers and after a risk exposure. The number of lifetime HIV tests performed was strongly associated with RHI in studies conducted in France [30] and Estonia [36]. The Singapore Health Promotion Board has been working with partner organizations to conduct programmes and campaigns targeted at high-risk individuals to urge them to go for regular HIV testing [47].

The HIV surveillance programmes in Singapore include anonymous testing, voluntary opt-out inpatient testing and antenatal screening. Table S2 shows the annual number of HIV tests and percentage tested positive for the three HIV surveillance programmes in 2013–2017. The proportion tested positive was highest at anonymous test sites compared with the other two HIV surveillance programmes, and it ranged from 1.0% (182 out of 17,781 tests done) in 2016 to 1.6% (227 out of 13,893 tests done) in 2013 [5]. Attendees at the Department of STI Control clinic, a specialist outpatient clinic for the diagnosis, treatment and control of STIs, constitute a sentinel population for unlinked HIV surveillance, and the HIV seroprevalence in this group ranged from 0.7% in 2013 to 1.5% in 2015 [5]. The surveillance of RHI is a useful additional tool to monitor ongoing HIV transmission in Singapore, as it sheds light on at-risk groups for which preventive efforts are targeted at. There have been several studies on late-stage HIV infection in Asian countries [8, 11], but estimation of the proportion of RHI was mostly limited to specific subpopulation groups such as MSM [40] and injecting drug users [48, 49]. In Singapore, the independent risk factors for late presentation to HIV care included older age at diagnosis and HIV detection via medical care [8], which are in contrast to those of RHI in this study.

The findings of this study should be examined in the light of its limitations. The observational design of our study precluded causal inference. A limitation inherent to routine surveillance databases is the self-reporting of epidemiological information such as exposure risk factors and HIV testing history prior to positive diagnosis. For newly-diagnosed HIV cases who had reported previous HIV test(s) prior to their positive diagnosis, we were unable to determine whether their infections were recently acquired as the date of their last negative HIV test was mostly unavailable. There may be additional unmeasured factors that could introduce confounding bias in our assessment of the association with RHI. As HIV diagnoses are subject to the number of persons tested and their testing patterns, there is a need to consider the estimated proportion of RHI in the context of frequencies of HIV testing and inter-test intervals in different subgroups [30, 50]. Although only 31.8% of all HIV cases notified to the National HIV Registry in 2013–2017 were included in this study, there were no major differences in the socio-demographic and epidemiological characteristics of those tested when compared with all the newly-diagnosed cases during the five-year period (Table 1).

Factors associated with misclassification by the BED-CEIA include long-term use of ART, low HIV viral load, and low CD4 cell count [51]. CD4 count can drop during sero-conversion [52], hence we might have slightly under-estimated the proportion of RHI for cases with CD4 count < 200 cells/mm^3^, as they would have been misclassified as NRHI according to the RITA in our study (Fig. 1). Nevertheless, the extent of misclassification was likely to be minimal with the use of the most widely used BED assay and additional consideration of clinical information. In addition to the use of BED-CEIA and false recency rate (FRR), other approaches such as antibody avidity tests or other detuned ELISA have been used in several studies to estimate recent infections alone or in combination [33, 34, 36, 38]. However, the principles of the three assays differ and the factors affecting FRR, as well as the window period during which the infection would be classified as recent, may also differ for each assay [20, 38]. Dual testing algorithms have been shown to reduce FRR, even without correction for late stages of disease [53]. There is potential to improve the surveillance of RHI by implementing serological tests with higher sensitivity and specificity to achieve a more accurate proportion of recent infections among newly-diagnosed HIV cases.

In conclusion, approximately one-fifth of newly diagnosed cases were diagnosed early. As only a minority of HIV infections were diagnosed at the early stage of the disease, there is a pressing need to increase the level of awareness of HIV/AIDS and encourage more at-risk individuals to go for early and regular HIV testing. The strong associations observed between modifiable behaviors (voluntary testing and HIV testing history) and RHI highlight the importance of HIV prevention and control strategies that increase the accessibility to HIV testing for at-risk groups in order to reduce ongoing transmission risk.

## Supplementary Information

**Additional file 1.** Supplementary Tables S1 and S2. All supplementary tables as listed in the article.

## Data Availability

The data that support the findings of this study are available from Li Wei Ang, National Public Health and Epidemiology Unit, National Centre for Infectious Diseases, but institutional restrictions apply to the availability of these data, which were used under license for the current study, and so are not publicly available.

## Abbreviations

aOR: Adjusted odds ratio
AfA: Action for AIDS
ART: Antiretroviral therapy
BED-CEIA: Biotinylated peptide-capture enzyme immunoassay
CI: Confidence interval
FRR: False recency rate
IDA: Infectious Diseases Act
IgG: Immunoglobulin
IQR: Interquartile range
MSM: Men having sex with men
NPHL: National Public Health Laboratory
NRHI: Non-recent HIV infection
RHI: Recent HIV infection
RITA: Recent infection testing algorithm
OD: Optical density
STIs: Sexually transmitted infections
WHO: World Health Organization
