# Supplementary material for: Epidemiological factors associated with recent HIV infection among newly-diagnosed cases in Singapore, 2013–2017
Source: BMC Public Health. 2021 Mar 2;21:430. doi: 10.1186/s12889-021-10478-5 (PMC7927232; doi:10.1186/s12889-021-10478-5)
Supplement: Supplementary file 1 — Additional file 1. Supplementary Tables S1 and S2. All supplementary tables as listed in the article. [file 12889_2021_10478_MOESM1_ESM.pdf]

Supplementary material

Epidemiological factors associated with recent HIV infection among newly-diagnosed cases in Singapore, 2013–2017

Li Wei Ang, Carmen Low, Chen Seong Wong, Irving Charles Boudville, Matthias Paul Han Sim Toh, Sophia Archuleta, Vernon Jian Ming Lee, Yee Sin Leo, Angela Chow, Raymond Tzer-Pin Lin

Table S1. Estimated proportion of recent HIV infection and associated risk factors among newly-diagnosed HIV-positive patients in Singapore and other countries

Country	Study subjects	Test and definition of RHI	Proportion of RHI (95% CI)	Independent risk factors
Singapore	701 HIV cases newly-diagnosed in 2013–2017, whose plasma samples were taken within one year from HIV diagnosis and before commencement of ART. The residual samples were tested for evidence of RHI as part of the national HIV molecular surveillance.	BED-CEIA assay was used.<br><br>Seropositive samples tested with normalized optical density (OD-n) <0.8 were initially classified as RHI. For those with low CD4 count (<200 cells/mm^3), they were reclassified as non-recent using RITA.	Overall: 19.0% (16.2%–22.0%)<br><br>Men: 19.1% (16.3%–22.3%)<br><br>MSM: 23.4% (19.6%–27.6%<br><br>Heterosexual men: 11.1% (7.6%–15.9%)<br><br>Women: 17.1% (8.5%–31.3%)	• Age 15-24 years at diagnosis vs ≥55 years<br>• Detection via voluntary testing compared with medical care<br>• History of HIV test(s) prior to positive diagnosis<br>• HIV diagnosis in the period after 2013–2014
Japan [1]	A total of 1,694 newly-diagnosed HIV cases who visited local health centers for voluntary counseling and testing in three metropolitan areas	LAg-Avidity Enzyme Immunoassay (EIA) was used.<br><br>RHI was defined as: (i) seronegative and nucleic acid	By metropolitan areas:<br>Tokyo<br>Mean: 38.6% (32.5%–44.8%)<br><br>Osaka<br>Mean: 30.1% (24.9%–35.3%)	

Country	Study subjects	Test and definition of RHI	Proportion of RHI (95% CI)	Independent risk factors
	in 2006–2015, Tokyo (1,105), Osaka (380) and Fukuoka (43). These HIV cases whose samples were used for recent infection assay, were ART-naive and did not have AIDS at diagnosis.	amplification test (NAT)-positive or (ii) seropositive and HIV-1 recent infection assay-positive.	Fukuoka<br>Mean: 20.4% (16.7%–24.0%)	
Taiwan [2]	683 HIV-positive newly diagnosed individuals reported to the Taiwan Centers for Disease Control during 2007-2009 were selected from different risk groups based on the specified proportion in each year.	BED-CEIA assay was used.<br><br>In confirmatory testing, specimens with OD-n ≤0.8 were considered a recent seroconversion. If OD-n >0.8, the specimen was considered a long term seroconversion.	Overall: 43.8%<br><br>MSM: 51.2%<br><br>Heterosexuals: 37.6%<br><br>Injecting drug users: 38.3%	
Australia [3]	All new HIV diagnoses reported by doctors and laboratories to state/territory health authorities.<br><br>Information on the date of the last negative or indeterminate test or date of onset of primary HIV has been routinely sought from each state/territory health jurisdiction since 1991.	Newly acquired HIV was defined as newly diagnosed infection with a negative or indeterminate HIV antibody test result or a diagnosis of primary HIV (seroconversion illness) within one year before HIV diagnosis.	By year:<br>2008: 31.5%<br>2011: 37.8%<br>2014: 39.2%<br>2017: 25.0%<br>2008–2017: 34.6%	

Country	Study subjects	Test and definition of RHI	Proportion of RHI (95% CI)	Independent risk factors
Australia [4]	437 MSM newly diagnosed with HIV infection at Melbourne Sexual Health Centre (MSHC) in Victoria, Australia between January 2007 and March 2016 were included.	Serological evidence of RHI was defined as a reactive screening test with a negative or indeterminate western blot in a patient in whom HIV diagnosis was confirmed through ancillary or repeat testing.<br>No serological evidence of recent HIV infection was defined as a reactive screening test with a positive western blot.	MSM: 26.8%<br><br>By time period:<br>2007–2009: 15.6%<br>2010–2012: 23.5%<br>2013–2014: 35.8%<br>2015–2016: 34.3%	
China [5]	Chinese MSM was recruited for a cross-sectional study in seven cities of China (Shanghai, Nanjing, Changsha, Zhengzhou, Ji'nan, Shenyang and Kunming) between June 2012 and June 2013,<br><br>444 participants tested positive for HIV, and their specimens were then tested for RHI.	BED-CEIA assay was used.<br><br>Specimens with initial OD-n >1.2 were classified as established HIV infection. Specimens with initial OD-n <1.2 were tested in triplicate to confirm their OD-n values. If the median OD-n value from all three tests was <0.8, the HIV case was considered as recently infected.	MSM: 41.9 % (37.3%–46.5%)	
England, Wales and Northern Ireland [6]	6,966 HIV cases newly-diagnosed in 2009–2011, whose samples were taken within four	Avidity index assay, based on detection of low-avidity antibodies, was used.	Overall: 14.7%<br><br>MSM: 22.3%<br><br>Heterosexuals: 7.8%	Among MSM<br>• Younger age groups: 15–24 years and 25–34

Country	Study subjects	Test and definition of RHI	Proportion of RHI (95% CI)	Independent risk factors
	months of the diagnosis date.	Avidity index value <0.8 was classified as RHI. Results with index values between 0.75 and 0.85 were retested and the mean of the two results was used.<br><br>Individuals with a CD4 count <200 cells/mm^3, diagnosis of an AIDS-defining illness or antiretroviral treatment before or at the time the sample was taken were re-classified as having long-term infections.	Injecting drug users: 5.6%	years vs ≥50 years<br>• UK as the probable country of infection vs abroad<br><br>Among heterosexuals<br>• 'Black other' ethnicity vs White<br>• UK as the probable country of infection vs abroad
United States [7]	964 patients whose HIV diagnosis was made within the past 12 months and who had never received antiretroviral therapy, were enrolled consecutively from HIV care clinics, HIV counseling and testing sites, and other clinical settings in 10 cities in 1997–2001 with specimens from those who had given consent.	Specimens were tested using a modified version of the HIV-1 enzyme immunoassay in which the sample dilution and sample and conjugate incubation times were modified to render the test less sensitive.<br><br>A standard OD value <1.0 was defined as RHI. Persons with RHI were presumed to have seroconverted within the past 170 days (95% CI 144–200).	Overall: 19.8%<br><br>Men: 21.7%<br><br>MSM: 25.5%<br><br>Female: 14.6%	• MSM contact<br>• Recent diagnosis of urethral gonorrhoea
France [8]	10,855 newly diagnosed HIV cases reported between July 2003 and December 2006, whose	A single, indirect enzyme-linked immunosorbent assay had been developed to quantify antibodies toward	Overall: 23.1% (22.3%–23.9%)<br><br>Men: 27.8% (26.8%–28.9%)	• Younger age groups vs ≥50 years

Country	Study subjects	Test and definition of RHI	Proportion of RHI (95% CI)	Independent risk factors
	dried serum spots were taken at the same time as HIV diagnosis and notification.	four HIV-1 antigens: consensus peptides of the immunodominant epitope of gp41 (IDE), consensus V3 peptides, recombinant integrase, and recombinant p24 [9]. The assay was used to identify RHI that can be used on dried serum spots (DSS).<br><br>Patients known to have AIDS (information collected from the HIV reporting form) were classified as established infection whatever the result of the assay.	MSM: 42.8% (41.0%–44.6%)<br><br>Heterosexuals: 16.3% (15.4%–17.3%)<br><br>Women: 15.6% (14.5%–16.7%)	• HIV exposure via MSM contact<br>• French nationality<br>• High economic status<br>• Tested after a risk exposure<br>• Tested for HIV three or more times during their lifetime
Spain [10]	1,125 newly-diagnosed HIV cases aged ≥16 years from participating centres, which represented about 50% of all the new HIV notifications collected by the voluntary reporting system in 2006–2008, whose samples were obtained within the first 6 months of diagnosis.	Two different tests for recent infection (TRI) were used: the Vironostika-LS assay (January 2006–May 2007) and the BED-CEIA (June 2007 onwards).<br><br>Patients whose samples tested positive in the TRI were considered as having RHI.	Overall: 23.0% (20.6%–25.6%)<br><br>Men: 24.6%<br><br>MSM: 31.1%<br><br>Heterosexuals: 15.4%<br><br>Women: 16.8%	• HIV exposure via MSM contact compared with heterosexual contact<br>• Age <30 years compared with >50 years
Sweden [11]	767 patients diagnosed with HIV in 2003–2010, whose plasma samples were included if they:	HIV-1 IgG capture BED enzyme-linked immunoassay was used.	Overall: 35%<br><br>MSM: 45%	• Younger age<br>• HIV exposure via MSM contact

Country	Study subjects	Test and definition of RHI	Proportion of RHI (95% CI)	Independent risk factors
	(i) were reported to have been infected in Sweden or were born in Sweden and reported to have been infected abroad;<br>(ii) had a successful BED result from a sample collected within 120 days from the date of diagnosis;<br>(iii) had a CD4 count available from 120 days before to 120 days after the date of sampling for BED testing.	Patients were classified as having an early diagnosis if they had a BED assay result of OD-n value <0.8 in a blood sample drawn within 4 months from diagnosis provided that they did not have low CD4 count (<200 cells/μl), low viral load (<400 copies/ml), ongoing ART, and/or an AIDS-defining illness at diagnosis.	Heterosexuals: 21%<br><br>Injecting drug users: 27%	
Austria [12]	205 HIV cases newly diagnosed between January 2002 and October 2003, whose serum samples used for the first diagnosis of HIV infection by antibody testing were still available from various hospitals.	Avidity index assay, based on detection of low-avidity antibodies [13].<br><br>Avidity index value <0.8 was classified as RHI within the last 6 months.	Overall: 27.3%<br><br>MSM: 40.0%<br><br>Heterosexuals: 16.7%	
Germany [14]	10,257 cases newly diagnosed with HIV during 2008-2014, with one additional dried serum spots (DSS) or dried plasma spots (DPS) sample available from a subgroup of	BED-CEIA assay was used.<br><br>Seropositive samples tested with normalized optical density (OD-n) <0.8 were initially classified as RHI.<br>For those with AIDS-defining illness reported on the	Overall: 30.4%<br><br>MSM: 35.0%<br><br>Heterosexuals: 20.6%<br><br>Injecting drug users: 34.9%	• Age <25 years vs older age groups<br>• HIV exposure via MSM contact<br>• German nationality

Country	Study subjects	Test and definition of RHI	Proportion of RHI (95% CI)	Independent risk factors
	laboratories which were recruited using convenience sampling for the study.	notification form, they were reclassified as non-recent using RITA.		
Estonia [15]	228 individuals aged ≥18 years newly-diagnosed with HIV in January–December 2013, and linked to care who participated in the Estonian HIV database, with serum samples and available HIV viral load measurements.	A limiting antigen (LAg) avidity enzyme immunoassay was used.<br><br>An OD value <1.5 was classified as RHI. According to RITA, samples with a viral load <1000 HIV-1 RNA copies/mL and individuals presenting with AIDS were reclassified as long-term infection.	Overall: 36%	• Repeat tester vs first-time tester
Italy [16]	2,608 antiretroviral-naïve HIV-positive patients enrolled in the ICONA Foundation Study cohort during 1996-2014, with documented dates of HIV-negative and positive antibodies tests.	Date of HIV seroconversion was estimated as the midpoint between the last available HIV-negative and the first available HIV-positive test.<br>Patients with estimated date of HIV seroconversion recorded within one year (since the last negative HIV serological test) were defined as RHI.	Overall: 37.6%	• Younger age at HIV diagnosis<br>• Higher baseline CD4+ T-cells<br>• Higher baseline HIV-RNA<br>• More recent calendar period (2010–2014) vs 1996–2000
Poland [17]	95 HIV-positive newly-diagnosed individuals who presented for HIV testing in 2006 at any site within the network of	Three RITA assays were used on all samples:<br>i) Avidity index assay, based on detection of low-avidity	Overall: 47.3% (any of the three RITA assays)	• Having two or more sexual partners in the past 12 months

Country	Study subjects	Test and definition of RHI	Proportion of RHI (95% CI)	Independent risk factors
	Voluntary Counseling and Testing (VCT) run by the National AIDS Centre, whose demographic and behavioural data were linked.	antibodies. Avidity index value <0.8 was identified as RHI.<br>ii) Detuned assay (less sensitive ELISA) based on low titers of HIV antibodies. Specimens that were non-reactive on the less sensitive assay were considered to be obtained from those with RHI.<br>iii) BED EIA HIV-1 incidence test based on a low ratio of HIV-1-specific IgG to total IgG in the sample. OD-n ≤0.8 was identified as RHI.		

ART, antiretroviral therapy; BED-CEIA, BED HIV-1 capture enzyme immunoassay; CI, confidence interval; MSM, men having sex with men; RHI, recent HIV infection; OD-n, normalized optical density; RITA, recent infection testing algorithm; STD, sexually transmitted disease.

References

1. Matsuokaa S, Nagashimab M, Sadamasub K, Moric H, Kawahatac T, Zaitsud S, et al. Estimating HIV-1 incidence in Japan from the proportion of recent infections. Prev Med Rep. 2019;16:100994.
2. Kao CF, Chang SY, Hsia KT, Chang FY, Yang CH, Liu HR, et al. Surveillance of HIV type 1 recent infection and molecular epidemiology among different risk behaviors between 2007 and 2009 after the HIV type 1 CRF07_BC outbreak in Taiwan. AIDS Res Hum Retroviruses. 2011;27(7):745-749.
3. Kirby Institute. HIV in Australia: annual surveillance short report 2018. Sydney: Kirby Institute, UNSW Sydney; 2018 [Cited 2020 Sep 20]. Available from: https://kirby.unsw.edu.au/sites/default/files/kirby/report/supplHIV2018_content_20180920r.pdf. Accessed 20 September 2020.
4. Medland NA, Nicholson S, Chow EPF, Read TRH, Bradshaw CS, Denham I, et al. Time from HIV infection to virological suppression: dramatic fall from 2007 to 2016. AIDS. 2017;31(17):2377-2385.
5. Xu JJ, Tang WM, Zou HC, Mahapatra T, Hu QH, Fu GF, et al High HIV incidence epidemic among men who have sex with men in China: results from a multi-site cross-sectional study. Infect Dis Poverty. 2016;5(1):82.

6. Aghaizu A, Murphy G, Tosswill J, DeAngelis D, Charlett A, Gill ON, et al. Recent infection testing algorithm (RITA) applied to new HIV diagnoses in England, Wales and Northern Ireland, 2009 to 2011. Euro Surveill. 2014;19(2):20673.
7. Schwarcz S, Weinstock H, Louie B, Kellogg T, Douglas J, Lalota M, et al. Characteristics of persons with recently acquired HIV infection: Application of the serologic testing algorithm for recent HIV seroconversion in 10 US cities. J Acquir Immune Defic Syndr. 2007;44(1):112-115.
8. Semaille C, Cazein F, Pillonel J, Lot F, Le Vu S, Pinget R, et al. Four years of surveillance of recent HIV infections at country level, France, mid 2003–2006: Experience and perspectives. Euro Surveill. 2008;13(36). pii: 18968.
9. Barin F, Meyer L, Lancar R, Deveau C, Gharib M, Laporte A, et al. Development and validation of an immunoassay for identification of recent human immunodeficiency virus type 1 infections and its use on dried serum spots. J Clin Microbiol. 2005;43(9):4441-4447.
10. Romero A, González V, Esteve A, Martró E, Matas L, Tural C, et al; AERI Study group. Identification of recent HIV-1 infection among newly diagnosed cases in Catalonia, Spain (2006-08). Eur J Public Health. 2012;22(6):802-808.
11. Widgren K, Skar H, Berglund T, Kling AM, Tegnell A, Albert J. Delayed HIV diagnosis common in Sweden, 2003-2010. Scand J Infect Dis. 2014;46(12):862-867.
12. Puchhammer-Stöckl E, Schmied B, Rieger A, Sarcletti M, Geit M, Zangerle R, et al. Low proportion of recent human immunodeficiency virus (HIV) infections among newly diagnosed cases of HIV infection as shown by the presence of HIV-specific antibodies of low avidity. J Clin Microbiol. 2005;43(1):497-498.
13. Suligoi B, Galli C, Massi M, Di Sora F, Sciandra M, Pezzotti P, et al. Precision and accuracy of a procedure for detecting recent human immunodeficiency virus infections by calculating the antibody avidity index by an automated immunoassay-based method. J Clin Microbiol. 2002;40(11):4015-4020.
14. Hofmann A, Hauser A, Zimmermann R, Santos-Hövener C, Bätzing-Feigenbaum J, Wildner S, et al. Surveillance of recent HIV infections among newly diagnosed HIV cases in Germany between 2008 and 2014. BMC Infect Dis. 2017;17(1):484.
15. Soodla P, Simmons R, Huik K, Pauskar M, Jõgeda EL, Rajasaar H, et al; Concerted Action on SeroConversion to AIDS and Death in Europe (CASCADE) Collaboration in EuroCoord. HIV incidence in the Estonian population in 2013 determined using the HIV-1 limiting antigen avidity assay. HIV Med. 2018;19(1):33-41.
16. Nozza S, Cozzi-Lepri A, Bai F, Rusconi S, Gori A, Cinque P, et al. Proportion and factors associated with recent HIV infection in a cohort of patients seen for care in Italy over 1996-2014: Data from the ICONA Foundation Study cohort. PLoS One. 2017;12(12):e0189045.
17. Rosińska M1, Marzec-Bogustawska A, Janiec J, Smoleń-Dzirba J, Wąsik T, Gniewosz J, et al; CASCADE Collaboration In Eurocoord. High percentage of recent HIV infection among HIV-positive individuals newly diagnosed at voluntary counseling and testing sites in Poland. AIDS Res Hum Retroviruses. 2013;29(5):805-813.

Table S2. Number of HIV tests done and percentage tested HIV positive for three HIV surveillance programmes in Singapore, 2013–2017

Year	HIV surveillance programme					
	Anonymous testing		Voluntary opt-out inpatient HIV testing		Antenatal screening	
	Total number of tests	% tested positive	Total number of tests	% tested positive	Total number of tests	% tested positive
2013	13,893	1.63%	33,297	0.12%	38,088	0.03%
2014	15,950	1.27%	30,834	0.19%	38,679	0.05%
2015	15,641	1.43%	30,123	0.16%	33,945	0.06%
2016	17,781	1.01%	28,684	0.17%	27,498	0.04%
2017	17,363	1.05%	28,724	0.20%	27,347	0.05%

Source: Ministry of Health, Singapore. Communicable Diseases Surveillance in Singapore 2017. Communicable Diseases Division, Ministry of Health, Singapore, 2018. Available from: https://www.moh.gov.sg/resources-statistics/reports/communicable-diseases-surveillance-in-singapore-2017. Accessed 24 July 2018.
